# Incidence of penile cancer worldwide: systematic review and meta-analysis

**DOI:** 10.26633/RPSP.2017.117

**Published:** 2017-11-30

**Authors:** Carlos Eduardo Montes Cardona, Herney Andrés García-Perdomo

**Affiliations:** 1 Department of Urology, Universidad del Valle Department of Urology, Universidad del Valle Santiago de Cali Colombia Department of Urology, Universidad del Valle, Santiago de Cali, Colombia.

**Keywords:** Penile neoplasms, incidence, observational studies, Neoplasias del pene, incidencia, estudios observacionales, Neoplasias penianas, incidência, estudos observacionais

## Abstract

**Objective.:**

*To determine the global incidence of penile cancer*.

**Methods.:**

*A systematic review and meta-analysis of observational studies was performed, with no limits on their language of publication. Analyses were performed using Stata 13 statistical software. A random-effects model was used, according to the heterogeneity found in the studies. The main outcome was expressed in terms of age-standardized incidence*.

**Results.:**

*A total of 23 studies were eligible, with 71 156 penile cancer patients in 86 countries. According to the review conducted, the estimated age-standardized incidence of penile cancer worldwide is 0.84 cases per 100 000 person-years (95% confidence interval: 0.79–0.89). Romania reported the highest incidence, 7.26 per 100 000 person-years, between 1983 and 1987; however, some countries in Latin America and Africa reported an incidence of between 2.0 and 5.7 per 100 000*.

**Conclusions.:**

*Penile cancer is considered a rare malignancy due to its already-known, particularly low incidence rate. The estimated age-standardized incidence rate by the world standard population today is 0.84 cases per 100 000 person-years. There were no significant differences in the incidence rate of penile cancer with respect to the distribution by continent or the trend over time*.

## RESUMEN

**Objetivo.:**

Determinar la incidencia mundial del cáncer de pene.

**Métodos.:**

Se realizó una revisión sistemática y un metanálisis de estudios de observación, sin ninguna limitación por el idioma de publicación. Para los análisis se usó el software estadístico Stata 13. Se usó un modelo de efectos aleatorios, conforme a la heterogeneidad encontrada en los estudios. El resultado principal se expresó en términos de incidencia ajustada por la edad.

**Resultados.:**

En total, 23 estudios (con una población total de 71 156 pacientes con cáncer de pene en 86 países) cumplieron con los criterios establecidos. Según esta revisión, se estima que la incidencia ajustada por la edad del cáncer de pene a escala mundial es de 0,84 casos por 100 000 años-persona (intervalo de confianza de 95%: 0,79–0,89). Rumania notificó la incidencia más alta, de 7,26 por 100 000 años-persona, entre 1983 y 1987; sin embargo, algunos países de América Latina y África notificaron una incidencia de entre 2,0 y 5,7 por 100 000.

**Conclusiones.:**

Se considera que el cáncer de pene es una neoplasia maligna rara debido a que la tasa de incidencia conocida es particularmente baja. Hoy en día, la tasa de incidencia ajustada por la edad estimada para la población estándar mundial es de 0,84 casos por 100 000 años-persona. No se observó ninguna diferencia significativa de la tasa de incidencia del cáncer de pene con respecto a la distribución por continente o la tendencia con el transcurso del tiempo.

**Objetivo.:**

Determinar a incidência mundial de câncer de pênis.

**Métodos.:**

Um estudo de revisão sistemática e meta-análise de estudos observacionais foi conduzido, sem restrição do idioma da publicação. As análises foram realizadas com o uso do software de estatística Stata 13. Um modelo de efeitos aleatórios foi usado, segundo a heterogeneidade encontrada nos estudos. O desfecho principal foi expresso como taxa de incidência padronizada por idade.

**Resultados.:**

Vinte e três estudos satisfizeram os critérios, englobando 71.156 pacientes com câncer de pênis em 86 países. De acordo com a revisão conduzida, a taxa de incidência padronizada por idade estimada para o câncer de pênis em todo o mundo é de 0,84 caso por 100.000 pessoas-ano (intervalo de confiança de 95% 0,79–0,89). A Romênia registrou a taxa de incidência mais elevada, 7,26 por 100.000 pessoas-ano, entre 1983 e 1987. Porém, alguns países da América Latina e África registraram taxas de incidência entre 2,0 e 5,7 por 100.000 pessoas-ano.

**Conclusões.:**

O câncer de pênis é uma neoplasia maligna rara devido à sua conhecida taxa de incidência especialmente baixa. A atual taxa de incidência padronizada por idade estimada para a população mundial padrão é de 0,84 caso por 100.000 pessoas-ano. Não foi encontrada diferença significativa na taxa de incidência do câncer de pênis quanto à distribuição por continente ou tendência temporal.

Penile cancer is a rare malignancy, especially in developed countries, in which the annual incidence is below 1 case per 100 000 men. This incidence represents less than 1% of malignancies in this gender; however, in certain Asian, African, and South American countries, the occurrence may represent up to 10% of cases (1). One of the countries with the highest incidence of penile cancer in the world is India, with rates up to 3.32 per 100 000 men in some regions. In contrast, rates among Jewish men born in Israel are reportedly very close to zero (2).

Penile cancer typically affects older men, and its incidence rate consistently increases with age (3). The age group most commonly affected by penile cancer is between 50 and 70 years, with a mean age of 67 years in the United States of America (4), although the disease has also been observed in patients under 40 years (3). The vast majority of patients have an apparently localized disease at diagnosis, with high-risk characteristics for nodal involvement but without clinical evidence of such involvement. This suggests that the morbidity and mortality among these patients is underestimated (5).

The risk factors associated with a greater likelihood of developing the disease, as well as the factors associated with higher incidence rates, are clearly known (6). Nevertheless, neither the global impact of penile cancer nor the variations according to different geographic areas or the changing trends in different time intervals are precisely known. The objective of this systematic review was to determine the incidence of penile cancer in the global population.

## METHODS

We conducted a meta-analysis of observational studies, following the Meta-analysis of Observational Studies in Epidemiology (MOOSE) (7) and the Preferred Reporting Items for Systematic Reviews and Meta-Analyses (PRISMA) guidelines (8, 9). Details of the protocol for this systematic review were registered on PROSPERO and can be accessed at www.crd.york.ac.uk/PROSPERO/display_record.asp?ID=CRD42016052212.

### Sources and search strategy

We conducted a systematic search in the following electronic databases: Cochrane CENTRAL Register and Cochrane Prostatic Diseases and Urologic Cancers Group, MEDLINE, Embase, and LILACS. We searched for materials published between January 1980 and December 2016. In the searches, we did not place any limits on the language of publication.

The MEDLINE search, done via Ovid, was: (exp Penile Neoplasms/or (penil$ adj3 cancer$).mp. or (penil$ adj3 tumor$).mp) and (Exp Morbidity/or exp Mortality/or exp incidence/or Burden.mp) and (exp Epidemiologic Studies/or (cross*sectional$ adj3 stud$).mp or (cohort$ adj3 stud$)). The Embase search was: (‘penis cancer’/exp or (penil* NEXT/3 neoplasm*):ti,ab or (penil* NEXT/3 cancer*):ti,ab or (penil* NEXT/3 tumor*):ti,ab) and (‘morbidity’/exp or ‘mortality’/exp or burden:ti,ab or ‘epidemiology’/exp or ‘incidence’/exp) and (‘cross-sectional study’/exp or ‘cohort analysis’/exp). The LILACS search was: (MH:“Neoplasias del pene”/or TW: “cancer pene$” or TW: “Tumor pene$”) and (MH: “Morbilidad”/or MH: “Mortalidad”/or MH: “incidencia”/and (MH: “Estudios epidemiológicos”/or MH: “Estudio observacional”/or MH: “Estudios de cohortes”)).

We also conducted a generic and academic Internet search and a metasearch. A search strategy defined for “gray literature” was included, to gather information from relevant sources, such as national ministries of health, the Pan American Health Organization (PAHO), hospital reports, databases of regional registries, congress proceedings, doctoral dissertations, reference lists of included studies, and consultations with experts and institutions related to the topic.

Another search source we used was the International Agency for Research on Cancer (IARC/GLOBOCAN). We also performed a search defined through the cancer population registry tool of Nordic countries, NORDCAN. We used the GLOBOCAN and NORDCAN information only when we did not find data to calculate the incidence in terms of age-standardized rate by world standard population (ASR-W).

We included observational studies, such as cohort studies, cases and controls, cross-sectional studies, case series, epidemiological surveillance studies, and official reports from national health ministries and from the World Health Organization (WHO), PAHO, and other specialized organizations. This was done through the various platforms mentioned, again without any restrictions on the language of publication.

We included studies regardless of the number of reported cases. Studies that reported the incidence of penile carcinoma in patients of any age, from any place in the world, between January 1980 and December 2016 were included. Studies were included that directly or indirectly reported the changing trend of the incidence rate of penile carcinoma. Studies that did not have documentation on the incidence rate of penile carcinoma and those without confirmation of disease pathology were excluded.

### Study selection

We two authors both independently reviewed the titles and abstracts of all citations identified and selected all of the potentially eligible studies. We two also independently evaluated the complete text versions of these articles, to determine whether each study fulfilled the inclusion criteria.

### Information extraction and data management

We two authors independently extracted the data using a standard electronic format that we had designed during the planning of the systematic review. The data extracted included name of the first author; year of publication; country; year of study; type of study; size of sample; and sociodemographic and clinical variables, such as age, penile lesion location, histopathological penile cancer type, clinical and pathological cancer staging, and circumcision status.

To resolve any disagreements regarding study eligibility, quality, and extracted data, we discussed the issue until we reached a consensus. In the case of unclear and/or incomplete information in the report of one of the studies, we contacted the study authors for clarification and to obtain the missing information.

### Assessment of bias risk in the included studies

We two authors both independently reviewed the reporting quality of all the studies, based on the Strengthening the Reporting of Observational studies in Epidemiology (STROBE) checklist (10); the results from the evaluation tools of a systematic review assessing the quality and susceptibility of bias in observational studies (11); and guidelines for evaluating medical research published by Fowkes et al. (12). We modified the STROBE checklist to include 15 relevant evaluation items, taking into account the nature of the potentially eligible studies, given that the checklist was designed for all types of observational studies and not specifically for epidemiological studies. When we two authors disagreed on the issue of checklist-item fulfillment, those differences were resolved by reaching a consensus.

### Statistical analysis

We performed the analyses using Stata 13 statistical software (StataCorp, College Station, Texas, United States). A random-effects model was used according to the heterogeneity found in the studies. We expressed incidence in terms of age-standardized rate by world standard population (ASR-W) (13), calculated as follows: number of new cases in a given location in a given age group, gender, and time period, divided by mean estimated population for the same year for that age group and gender, multiplied by 100 000. The result was expressed as the number of new cases per 100 000 person-years. When the source population of each incidence-adjusted rate had not been published, the standardized population published by the WHO for the corresponding year of each study was extracted. A planned analysis of subgroups by continent and time interval was conducted to assess potential differences in the incidence rate in relation to these two factors. A summary report of penile carcinoma cases described in the included studies was generated. No sensitivity analysis was designed for this meta-analysis due to the expected and identified weight for each included study. An evaluation was performed to identify reporting or publication bias, using a funnel plot (14).

### Evaluation of heterogeneity

We assessed the heterogeneity among the studies by visual inspection of forest plots to determine whether the confidence intervals overlapped. Subsequently, an I2 test was performed. The I2 statistic describes the percentages of variability of effect estimates that are due to heterogeneity rather than sampling error (9). Values of 25%, 50%, and 75% in the I2 test correspond to low, medium, and high levels of heterogeneity, respectively.

### Subgroup analysis

We performed subgroup analysis according to geographical location, by continent. Additionally, we carried out a subgroup analysis by time intervals for studies reporting incidence rates before 1990, from 1990 to 1999, and after 1999.

## RESULTS

**FIGURE 1.**
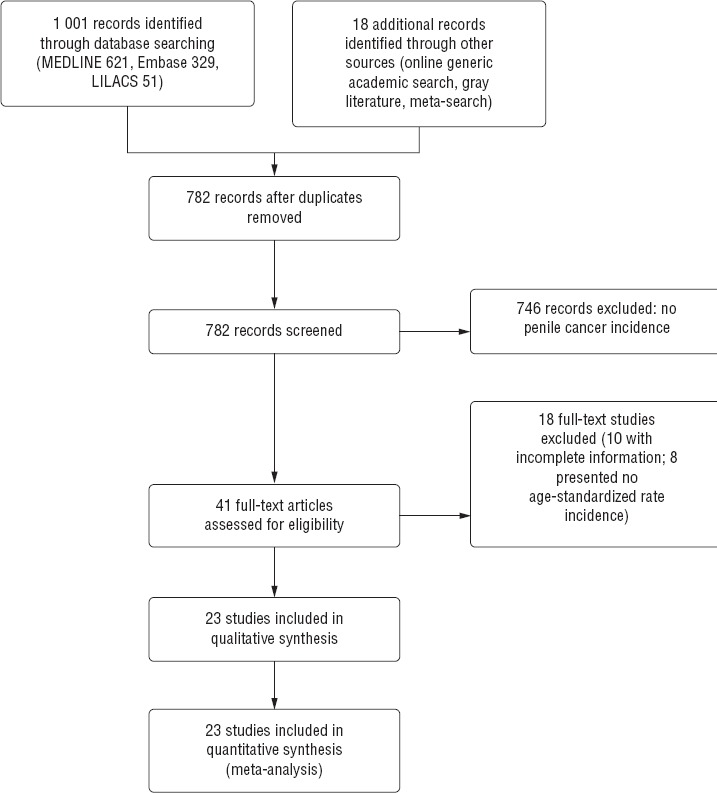
Flow chart of process for selecting studies used in determining the incidence of penile cancer in the global population

***Source:*** Prepared by the authors from the study data.

The study selection process is shown in Figure 1. The literature search strategy yielded 1 001 potentially eligible articles, including 621 MEDLINE articles (through Ovid), 329 Embase articles, and 51 LILACS articles. In addition, 18 related, potentially eligible epidemiological articles and reports were identified through the manual search and from references in the included articles. Out of this total number, 41 articles and reports were included for detailed assessment. Subsequently, 18 full-text articles (3, 15–31) as well as 5 reports from the International Agency for Research on Cancer (IARC) (32–36) fulfilled the inclusion criteria and were included in the meta-analysis. From these sources, the records of 71 156 cases of patients diagnosed with penile cancer, from a total of 86 countries, were collected.

By definition, all the included studies were observational and descriptive, as they were population studies of incidence. Therefore, the main characteristics shown for each of the included articles are: author, publication date, period covered, country, and continent. Although the proportion of original articles was greater, most of the records obtained came from 5 of the 10 volumes of the Cancer Incidence in Five Continents (CI5) series that the IARC has produced (Table 1). (IARC has also published a database compilation of the main CI5 volumes called CI5*plus,* and it was taken into account for this study, as can be seen in Table 1.)

**TABLE 1.** Characteristics of included studies

Author	Publication Date	Period covered	Country	Continent
Wabinga	2000	1967–1971	Uganda	Africa
Wabinga	2000	1991–1994	Uganda	Africa
Wabinga	2000	1995–1997	Uganda	Africa
Wabinga	2000	1960–1966	Uganda	Africa
Goodman	2007	1995–2003	USA	North America
Hernandez	2008	1998–2003	USA	North America
Jin	1999	1972–1974	Shanghai (China)	Asia
Van der Zwan	2013	2004–2008	Netherlands	Europe
Colon-Lopez	2012	1992–2004	Puerto Rico (USA)	Latin America
Goncalves da Fonseca	2010	1996–2006	Brazil	Latin America
Navarro M	2004	2002–2003	Chile	Latin America
Paredes C	1989	1982–1986	Dominican Republic	Latin America
Manit	2013	1979–2009	England	Europe
Constance J	2013	1978–2010	Denmark	Europe
Olsen J	2012	2004–2007	Denmark	Europe
Robinson D	2009	1960–2004	England	Europe
Kirrander P	2015	2000–2012	Sweden	Europe
Jan M	2015	2004–2008	Netherlands	Europe
Graafland	2010	1989–2006	Netherlands	Europe
Bray F	2012	1964–2003	Nordic countries	Europe
Vatanasapt	1995	1990	Thailand	Asia
Vatanasapt	1995	1983–1987	Singapore	Asia
Barnholtz-Sloan	2007	1973–2002	USA	North America
Jin	1999	1993–1994	Shanghai (China)	Asia
CI5*plus* database	2014	1980–1989	Australia	Oceania
CI5*plus* database	2014	1990–1999	Australia	Oceania
CI5*plus* database	2014	2000–2007	Australia	Oceania
CI5*plus* database	2014	1988–1989	Austria	Europe
CI5*plus* database	2014	1990–1999	Austria	Europe
CI5*plus* database	2014	2000–2007	Austria	Europe
CI5*plus* database	2014	1988–1989	Croatia	Europe
CI5*plus* database	2014	1990–1999	Croatia	Europe
CI5*plus* database	2014	2000–2007	Croatia	Europe
CI5*plus* database	2014	1980–1989	Denmark	Europe
CI5*plus* database	2014	1990–1999	Denmark	Europe
CI5*plus* database	2014	2000–2007	Denmark	Europe
CI5*plus* database	2014	1980–1989	Estonia	Europe
CI5*plus* database	2014	1990–1999	Estonia	Europe
CI5*plus* database	2014	2000–2007	Estonia	Europe
CI5*plus* database	2014	1980–1989	France	Europe
CI5*plus* database	2014	1990–1999	France	Europe
CI5*plus* database	2014	2000–2007	France	Europe
CI5*plus* database	2014	1980–1989	Iceland	Europe
CI5*plus* database	2014	1990–1999	Iceland	Europe
CI5*plus* database	2014	2000–2007	Iceland	Europe
CI5*plus* database	2014	1983–1987	Romania	Europe
CI5*plus* database	2014	1980–1989	Italy	Europe
CI5*plus* database	2014	1990–1999	Italy	Europe
CI5*plus* database	2014	2000–2007	Italy	Europe
CI5*plus* database	2014	1980–1989	Netherlands	Europe
CI5*plus* database	2014	1990–1999	Netherlands	Europe
CI5*plus* database	2014	2000–2007	Netherlands	Europe
CI5*plus* database	2014	1980–1989	Poland	Europe
CI5*plus* database	2014	1990–1999	Poland	Europe
CI5*plus* database	2014	2000–2006	Poland	Europe
CI5*plus* database	2014	1994–1999	Rusia	Europe
CI5*plus* database	2014	2000–2007	Rusia	Europe
CI5*plus* database	2014	1980–1989	Slovakia	Europe
CI5*plus* database	2014	1990–1999	Slovakia	Europe
CI5*plus* database	2014	2000–2007	Slovakia	Europe
CI5*plus* database	2014	1980–1989	Switzerland	Europe
CI5*plus* database	2014	1990–1999	Switzerland	Europe
CI5*plus* database	2014	2000–2007	Switzerland	Europe
CI5*plus* database	2014	1980–1989	Scotland	Europe
CI5*plus* database	2014	1990–1999	Scotland	Europe
CI5*plus* database	2014	2000–2007	Scotland	Europe
CI5*plus* database	2014	1980–1989	England	Europe
CI5*plus* database	2014	1990–1999	England	Europe
CI5*plus* database	2014	2000–2007	England	Europe
CI5*plus* database	2014	1993–1999	Ireland	Europe
CI5*plus* database	2014	2000–2007	Ireland	Europe
CI5*plus* database	2014	1980–1989	Spain	Europe
CI5*plus* database	2014	1990–1999	Spain	Europe
CI5*plus* database	2014	2000–2007	Spain	Europe
CI5*plus* database	2014	1980–1989	Brazil	Latin America
CI5*plus* database	2014	1990–1999	Brazil	Latin America
CI5*plus* database	2014	2000–2007	Brazil	Latin America
CI5*plus* database	2014	1985–1989	Ecuador	Latin America
CI5*plus* database	2014	1990–1999	Ecuador	Latin America
CI5*plus* database	2014	2000–2007	Ecuador	Latin America
CI5*plus* database	2014	1983–1989	Colombia	Latin America
CI5*plus* database	2014	1990–1999	Colombia	Latin America
CI5*plus* database	2014	2000–2007	Colombia	Latin America
CI5*plus* database	2014	1980–1989	Costa Rica	Latin America
CI5*plus* database	2014	1990–1999	Costa Rica	Latin America
CI5*plus* database	2014	2000–2007	Costa Rica	Latin America
CI5*plus* database	2014	1980–1989	Canada	North America
CI5*plus* database	2014	1990–1999	Canada	North America
CI5*plus* database	2014	2000–2007	Canada	North America
CI5*plus* database	2014	1980–1989	USA	North America
CI5*plus* database	2014	1990–1999	USA	North America
CI5*plus* database	2014	2000–2007	USA	North America
CI5*plus* database	2014	1980–1989	Hawaii (USA)	North America
CI5*plus* database	2014	1990–1999	Hawaii (USA)	North America
CI5*plus* database	2014	2000–2007	Hawaii (USA)	North America
CI5*plus* database	2014	1993–1999	Uganda	Africa
CI5*plus* database	2014	2000–2007	Uganda	Africa
CI5*plus* database	2014	1983–1989	Philippines	Asia
CI5*plus* database	2014	1990–1999	Philippines	Asia
CI5*plus* database	2014	2000–2007	Philippines	Asia
CI5*plus* database	2014	1980–1989	India	Asia
CI5*plus* database	2014	1990–1999	India	Asia
CI5*plus* database	2014	2000–2007	India	Asia
CI5*plus* database	2014	1980–1989	Israel	Asia
CI5*plus* database	2014	1990–1999	Israel	Asia
CI5*plus* database	2014	2000–2007	Israel	Asia
CI5*plus* database	2014	1980–1989	Japan	Asia
CI5*plus* database	2014	1990–1999	Japan	Asia
CI5*plus* database	2014	2000–2007	Japan	Asia
CI5*plus* database	2014	1980–1989	Singapore	Asia
CI5*plus* database	2014	1990–1999	Singapore	Asia
CI5*plus* database	2014	2000–2007	Singapore	Asia
CI5*plus* database	2014	1980–1989	Thailand	Asia
CI5*plus* database	2014	1990–1999	Thailand	Asia
CI5*plus* database	2014	2000–2007	Thailand	Asia

***Source:*** Prepared by the authors from the study data.

Most of the studies excluded during selection did not report the incidence rate for penile cancer or the data necessary for its calculation, as they were often formatted as narrative reviews of some characteristics of the disease.

### Reporting quality assessment

In general, according to the modified STROBE checklist that we used, the reporting quality of the original articles (not applied to the CI5 reports) was moderate, with an average of 11 of 15 (77%) items approved (i.e., relevant evaluation items fulfilled). There was no correlation between score and publication date. The numbers of approved/unapproved items of the STROBE reporting quality assessment are shown in Table 2.

**TABLE 2.** Risk of bias assessment

**STROBE REPORTING QUALITY**	1a	1b	2	3	4	5	6	7	8	11	12a	14a	15	18	19
NAVARRO M 2004		✓	✓	✓	✓	✓	✓	✓	✓			✓	✓	✓	
PAREDES C 1989			✓			✓									
MANIT 2013	✓	✓		✓	✓	✓	✓	✓	✓	✓	✓	✓	✓	✓	✓
OLSEN J 2012	✓	✓	✓	✓		✓	✓	✓		✓	✓		✓	✓	
CONSTANCE J 2013	✓	✓	✓	✓	✓	✓	✓	✓	✓	✓	✓		✓	✓	
ROBINSON D 2009	✓		✓	✓	✓	✓	✓	✓	✓	✓	✓		✓	✓	
KIRRANDER P 2015	✓	✓	✓	✓	✓	✓	✓	✓	✓	✓	✓	✓	✓	✓	
JAN M 2015	✓	✓	✓	✓	✓	✓	✓	✓	✓	✓		✓	✓	✓	✓
GRAAFLAND 2010	✓	✓	✓	✓	✓	✓	✓	✓	✓	✓	✓		✓	✓	
BRAY F 2012		✓		✓		✓	✓	✓	✓	✓			✓	✓	
VATANASAPT 1995		✓	✓	✓		✓	✓	✓		✓	✓		✓	✓	
COLON-LOPEZ 2012	✓	✓	✓	✓	✓	✓	✓	✓	✓	✓	✓	✓	✓	✓	
GONZALVES A 2010	✓	✓	✓	✓		✓	✓	✓	✓				✓	✓	✓
WABINGA HR 2000		✓	✓	✓		✓	✓	✓	✓				✓	✓	
BARNHOLTZ-SLOAN 2007	✓	✓	✓	✓	✓	✓	✓	✓	✓	✓	✓		✓	✓	✓
HERNANDEZ 2008	✓	✓	✓	✓	✓	✓	✓	✓	✓	✓	✓	✓	✓	✓	✓
JIN 1999			✓	✓		✓	✓	✓		✓			✓	✓	
GOODMAN 2007	✓	✓	✓	✓	✓	✓	✓	✓	✓	✓	✓		✓	✓	

***Source:*** Prepared by the authors from the study data.

### Results from individual studies

**FIGURE 2.**
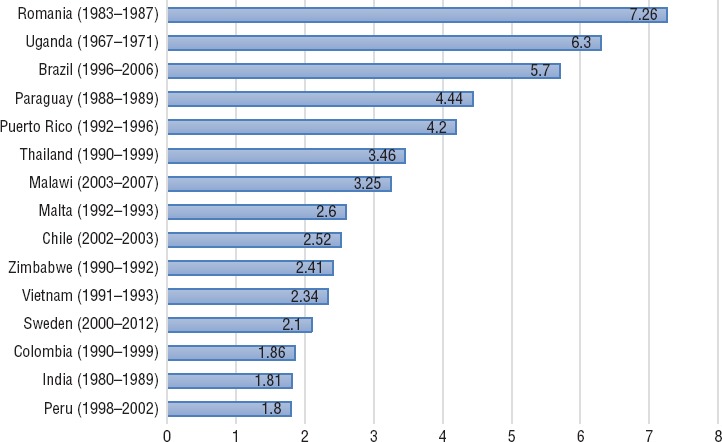
Countries with the highest incidence of penile cancer (age-standardized rate, cases per 100 000 person-years)

***Source:*** Prepared by the authors from the study data.

According to our review, the estimated incidence of the ASR of penile cancer worldwide is 0.84 cases per 100 000 person-years (95% confidence interval (CI): 0.79–0.89). The range was from 0.0 to > 2.0 cases per 100 000 person-years. The lower end of that range was more often seen in most of the Asian and non-sub-Saharan African countries. The highest incidence rate of penile cancer was reported in Romania between 1983 and 1987, with 7.26 cases per 100 000 person-years (Figure 2).

Among the 15 countries with the highest incidence of penile cancer, 3 of them were in Africa: Uganda, with an incidence of 6.3 cases per 100 000 person-years in the 1967–1971 period, followed by Malawi and Zimbabwe, with 3.25 and 2.41, respectively (Figure 2). Also among the 15 were 6 countries and territories from Latin America. Among those 6, Brazil reported the highest incidence (5.7 cases per 100 000 person-years), followed by Paraguay, Puerto Rico, and Chile. Also among the 15 were 3 Asian countries: Thailand, Vietnam, and India.

**FIGURE 3.**
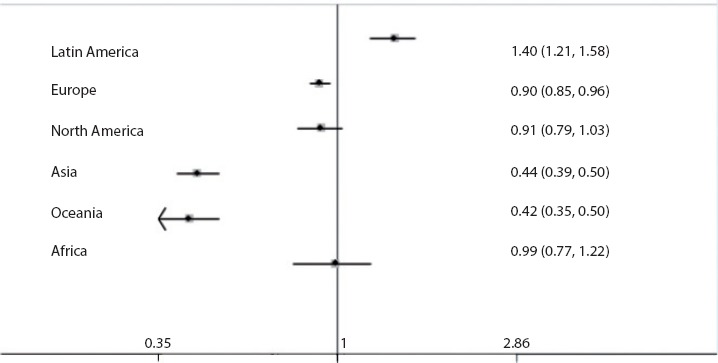
Incidence of penile cancer by geographic area, in terms of number of cases per 100 000 person-years, with 95% confidence interval

***Source:*** Prepared by the authors from the study data.

In the subgroup analysis of the five continents (six geographical zones, with the separation of the Americas into Latin America and North America), the results were: Europe, 0.90 cases per 100 000 person-years; Asia (which included Israel), 0.44 cases per 100 000 person-years; Africa, 0.99 cases per 100 000 person-years; Oceania, 0.42 cases per 100 000 person-years; Latin America, 1.40 cases per 100 000 person-years; and North America, 0.91 cases per 100 000 person-years (Figure 3). There were no significant differences in penile cancer incidence grouped by continent or period.

In the analysis conducted according to the year or the registration period of the incidence rate (for the studies that could be assigned to one of the periods), we found an ASR of 0.70 cases per 100 000 person-years (95% CI: 0.59–0.80) from 1980 to 1989, 0.94 cases per 100 000 person-years (95% CI: 0.84–1.04) from 1990 to 1999, and 0.89 cases per 100 000 person-years (95% CI: 0.80–0.99) from 2000 to 2009. There were no significant differences among the three periods.

**FIGURE 4.**
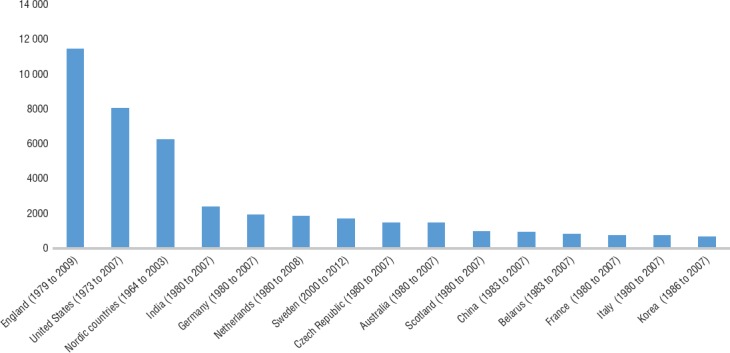
Number of cases of penile cancer reported for the countries with the highest incidence, for the indicated time periods

***Source:*** Prepared by the authors from the study data.

The period registered differed widely among the countries, ranging from 1 to 39 years, and from 1964 to 2012. The Nordic countries reported the longest period (1964–2003), followed by England and the United States. In terms of the number of cases, England reported the highest number, 11 478 (in a 30-year period), followed by United States (8 058 cases in 34 years), and the Nordic countries (6 272 cases in 39 years). Figure 4 presents the number of cases reported during a determined period for the 15 countries with the highest incidence of penile cancer.

## DISCUSSION

In our systematic review and meta-analysis, we found disparities and wide variations in terms of the incidence of penile cancer in each country over the past decades, with extreme values ranging from 0 up to 7.26 cases per 100 000 person-years. Considering that penile cancer has been classified as one of the so-called “rare malignancies” (mean incidence rates of less than 6 cases per 100 000 person-years), finding records of incidence rates much higher than previously documented is particularly striking (37, 38). However, our results are consistent with other reports of incidence rates close to 0, specifically in the Israeli-born Jewish population and in other Asian and African countries in which circumcision is a common procedure linked to religious practices. It is known that there is near to a 70% reduced risk of developing penile cancer throughout life in the circumcised population, especially when that procedure is performed close to birth (39). The circumcision effect could explain the incidence rates close to 0 found in these populations.

In contrast, never had such high rates been reported in countries such as Romania and other European nations, which prompts us to explore the nature of this isolated finding. The other locations with a noticeably high incidence of penile cancer are countries or territories whose information was documented in previous publications, with Uganda, Brazil, Puerto Rico, and Thailand leading the list (3, 4, 21, 31, 40).

Although the global incidence of penile cancer is considerably lower than in those high-rate countries, that worldwide incidence estimate is a result of the dilution effect of the large population considered. It is important to determine the cultural trends and lifestyles of each of the regions in order to establish the risk and protective factors that might explain the wide-ranging results reported in our study.

In terms of limitations with our review, it is possible that underreporting in developing countries is one of the more important ones. Another pronounced limitation could be that we completed information with the GLOBOCAN and NORDCAN registries when studies did not have adequate information to perform the analysis. However, it is important to note that the data coming from these registries are substantial, are collected in a standardized and trusted way, and contain precise information to use in calculating adjusted incidence rates.

Another limitation of this study was the impossibility of breaking down the results in such a way that we could calculate the accumulated risk and incidence per specific age group, the individual yearly results to represent the incidence trend accurately, and the average annual percentage change. However, it is clear that the incidence of penile cancer has remained stable over time, with no significant changes, according to our analysis of the three time periods.

A high-quality cancer population registry is a very important tool for all countries. However, this feature was absent in most of the reports we evaluated, due to the lack of consistent and steady reporting over time, which may limit the analysis in our systematic review and meta-analysis. Nevertheless, there are notable examples of high-quality registries, in countries such as England, the United States, and the Nordic nations. These three had the longest reporting periods and the highest number of cases, despite their relatively low disease incidence rates. Some reports made by independent researchers lack a reference population, and most of these investigators express incidence in terms of percentages or frequency, which limits overall estimates of penile cancer incidence. For example, Brazil and Paraguay reported 4.2 cases per 100 000 person-years in the late 1980s (41), but there were no further registries in the 1990s or 2000s that could help demonstrate a stable penile cancer incidence in these two countries.

This report is the first systematic review and meta-analysis in which the global incidence of penile cancer has been summarized. The report’s importance mainly lies in evaluating aspects of the geographic distribution and the dynamic behavior of penile cancer incidence over time. While being aware of the limitations in our study, we must still encourage surveillance systems around the world to collect and adequately report the incidence of penile cancer. In that work, there must be less information and selection bias, in order to seek and find public health interventions that could help prevent this catastrophic disease. Further studies are needed to establish relationships between potential risk factors and the incidence rates found in this review. Additional research is also needed on the interrelationship among other significant epidemiological features, such as associated mortality, the impact of treatment, and factors that may influence access to treatment.

### Conclusions

Penile cancer is considered a rare malignancy due to its already-known, particularly low incidence rate. The age-standardized incidence rate by the world standard population today is 0.84 cases per 100 000 person-years. This rate is a synthesis of values that are influenced by the socioeconomic and cultural factors of different geographic areas, including religious practices. This is particularly true with circumcision, which is more common in many Asian and African countries.

There were no significant differences in penile cancer incidence grouped by continent or period. It’s important to conduct further studies and improve penile cancer reporting in order to determine the factors that may influence differences in the incidence of penile cancer among countries around the world.

#### Disclaimer.

Authors hold sole responsibility for the views expressed in the manuscript, which may not necessarily reflect the opinion or policy of the *RPSP/PAJPH* or PAHO.
